# Supplementary figures and images for: Analysis of postoperative intraocular pathologies in patients with mature cataracts
Source: PLoS One. 2022 Jan 31;17(1):e0263352. doi: 10.1371/journal.pone.0263352 (PMC8803149; doi:10.1371/journal.pone.0263352)

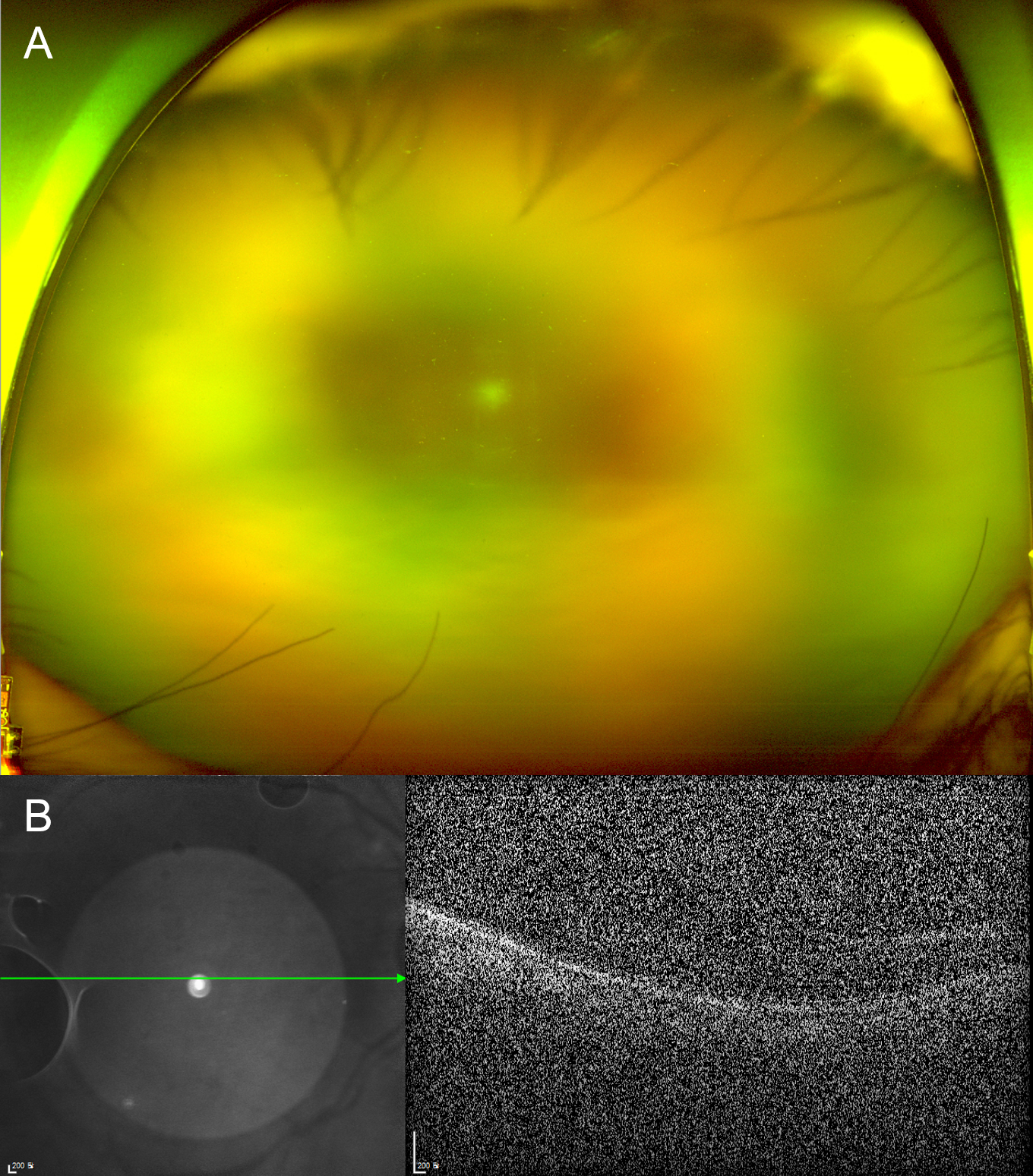

Supplement: S1 Fig — A. Wide fundus photography showing indistinguishable retinal structures. B. Only the retinal pigment epithelium layer is barely observed in the OCT image with an image quality of 5. OCT, optical coherence tomography. (TIF) [file pone.0263352.s001.tif]
