# Supplementary material for: Analysis of postoperative intraocular pathologies in patients with mature cataracts
Source: PLoS One. 2022 Jan 31;17(1):e0263352. doi: 10.1371/journal.pone.0263352 (PMC8803149; doi:10.1371/journal.pone.0263352)
Supplement: Attachment — Submitted filename: Response to Reviewers.docx [file pone.0263352.s003.docx]

November 22, 2021

Michael Mimouni

Academic Editor

*PLOS ONE*

Dear Editors,

We would like to resubmit the attached manuscript entitled “**Analysis of postoperative intraocular pathologies in patients with mature cataracts**” (manuscript ID: PONE-D-21-32917) for publication as a research article in the *PLOS ONE*.

We appreciate the reviewers' comments and suggestions, which have helped to improve the article tremendously*.* We have made appropriate corrections accordingly. Our point-by-point responses to the comments follow this letter.

Each of the coauthors has seen and agrees with each of the changes made to the revised manuscript and to the way his name is listed. Further, this manuscript has been checked by a professional English editor. Please find herewith all the required files and forms.

Data in this study cannot be made publicly available for ethical restrictions. The Public Institutional Bioethics Committee, designated by the South Korea Ministry of Health and Welfare, has placed ethical restrictions on the sharing of the data underlying this study in order to protect patient identities. Thus, the data are only available on request. In order to request the minimal data set, please contact the IRB office. (phone: 82-2-737-8959; email: irb@nibp.kr)

The page and line numbers mentioned in this response letter refer to the ‘revised manuscript with track changes’ file.

We hope that the revised manuscript will be considered suitable for publication in the *PLOS ONE*.

Sincerely,

**Kwan Hyuk Cho, MD, MSc**

Retina Center, Moon’s Eye Clinic, 762 Jeongjo-ro, Paldal-gu, Suwon-si, Gyeonggi-do, 16262, South Korea

Tel: +82-31-251-1373, Fax: +82-31-244-3046, E-mail: whmed@hanmail.net **Journal Requirements:**

When submitting your revision, we need you to address these additional requirements.

1.Please ensure that your manuscript meets PLOS ONE's style requirements, including those for file naming. The PLOS ONE style templates can be found at

https://journals.plos.org/plosone/s/file?id=wjVg/PLOSOne_formatting_sample_main_body.pdf and

https://journals.plos.org/plosone/s/file?id=ba62/PLOSOne_formatting_sample_title_authors_affiliations.pdf

**Answer:** We thank you for the opportunity to submit a revised version of the manuscript. As per your comments, we have checked that our manuscript meets the journal’s style requirements in the pdf files above.

2. Please review your reference list to ensure that it is complete and correct. If you have cited papers that have been retracted, please include the rationale for doing so in the manuscript text, or remove these references and replace them with relevant current references. Any changes to the reference list should be mentioned in the rebuttal letter that accompanies your revised manuscript. If you need to cite a retracted article, indicate the article’s retracted status in the References list and also include a citation and full reference for the retraction notice.

**Answer:** We have checked that the reference list is complete and correct.

3. In your Data Availability statement, you have not specified where the minimal data set underlying the results described in your manuscript can be found. PLOS defines a study's minimal data set as the underlying data used to reach the conclusions drawn in the manuscript and any additional data required to replicate the reported study findings in their entirety. All PLOS journals require that the minimal data set be made fully available. For more information about our data policy, please see http://journals.plos.org/plosone/s/data-availability.

Upon re-submitting your revised manuscript, please upload your study’s minimal underlying data set as either Supporting Information files or to a stable, public repository and include the relevant URLs, DOIs, or accession numbers within your revised cover letter. For a list of acceptable repositories, please see http://journals.plos.org/plosone/s/data-availability#loc-recommended-repositories. Any potentially identifying patient information must be fully anonymized.

Important: If there are ethical or legal restrictions to sharing your data publicly, please explain these restrictions in detail. Please see our guidelines for more information on what we consider unacceptable restrictions to publicly sharing data: http://journals.plos.org/plosone/s/data-availability#loc-unacceptable-data-access-restrictions. Note that it is not acceptable for the authors to be the sole named individuals responsible for ensuring data access.

We will update your Data Availability statement to reflect the information you provide in your cover letter.

**Answer:** Data in this study cannot be made publicly available for ethnic restrictions. Therefore, we have addressed them in detail in the revised cover letter. We also have provided contact information for ethics committee to which data requests may be sent.

4. PLOS requires an ORCID iD for the corresponding author in Editorial Manager on papers submitted after December 6th, 2016. Please ensure that you have an ORCID iD and that it is validated in Editorial Manager. To do this, go to ‘Update my Information’ (in the upper left-hand corner of the main menu), and click on the Fetch/Validate link next to the ORCID field. This will take you to the ORCID site and allow you to create a new iD or authenticate a pre-existing iD in Editorial Manager. Please see the following video for instructions on linking an ORCID iD to your Editorial Manager account: https://www.youtube.com/watch?v=_xcclfuvtxQ

**Answer:** The ORCID iD of the corresponding author has been linked. Thank you.

**Reviewers' comments:**

Reviewer #1: Well written and simple article. Conclusions are not surprising. I wonder if the cataract surgery itself could have affected the post op findings (ERM, or retinal tears?). In any event I do not suppose this to carry clinical significance.

**Answer:** Thank you for your thoughtful comments. As suggested, we reviewed the chart of all patients again to find any post op findings. During the mean follow-up period of 126 days, 5 cases of elevated intraocular pressure and 1 case of cystoid macular edema were noted postoperatively. Otherwise, no cases with epiretinal membrane, endophthalmitis, or retinal detachment were found.

As the reviewer commented, we also hypothesized that the prevalence of intraocular pathologies would not be particularly high compared to that of normal population. However, we thought our findings are significant because this is the first report to quantify the frequency and odds ratio of the intraocular pathologies observed after mature cataract surgery. Although our study may not carry great clinical significance, it will be helpful to explain the risks of intraocular pathologies in detail by presenting evidence in this study rather than vague warnings for patients with mature cataracts. We added this point in the result and conclusion section.

In page 9, line 109.

During the mean follow-up period of 126 days, 5 cases of elevated intraocular pressure and 1 case of cystoid macular edema were noted postoperatively. Otherwise, no cases with epiretinal membrane, endophthalmitis, or retinal detachment were found.

In page 11, line 151.

Our results would help patients with mature cataract understand the risk of intraocular pathologies with obvious evidence.

Reviewer #2: This is an interesting paper regarding the prevalence and risk factors of intraocular pathologies after mature cataract surgery. I have few questions for the authors:

1.- Although you state in your discussion that limited posterior segment pathologies can be seen on ocular ultrasonography, don’t you think that it is an important study to perform in all patients with dense cataract in which the fundus examination is not possible?

You report that one eye had a retinal detachment (RD) after cataract surgery, in this case, it would be important to have a previous ocular ultrasound, because you do not know if the RD was a postoperative complication of the surgery or it was already present before the surgery.

**Answer:** We totally agree with you that ocular ultrasonography still has an important meaning for preoperative evaluation in eyes with mature cataract. As you commented, we added this point in the discussion section.

In page 10, line 128,

However, this difference could not diminish the clinical implication of preoperative evaluation using ultrasonography in eyes with mature cataract. This is because, even though the frequency is small, it is possible to detect diseases with great clinical significance, such as one case of retinal detachment found in this study, using ultrasonography.

2.- You report that 73% of the eyes had the same pathology in the fellow eye, could you please specify which pathologies were found binocularly?

**Answer:** We apologize for insufficient explanation regarding the results. As your suggestion, we mentioned specific results about eyes that had the same pathology in the fellow eye.

In page 7, line 89,

7 with drusen, 5 with diabetic retinopathy, 4 with myopic degeneration, 3 with glaucoma, 2 with lattice degeneration and exudative age-related macular degeneration, respectively, 1 with epiretinal membrane, branch retinal vein occlusion, peripheral chorioretinal atrophy, and retinal tear, respectively.

While revising your submission, please upload your figure files to the Preflight Analysis and Conversion Engine (PACE) digital diagnostic tool, https://pacev2.apexcovantage.com/. PACE helps ensure that figures meet PLOS requirements. To use PACE, you must first register as a user. Registration is free. Then, login and navigate to the UPLOAD tab, where you will find detailed instructions on how to use the tool. If you encounter any issues or have any questions when using PACE, please email PLOS at figures@plos.org. Please note that Supporting Information files do not need this step.

**Answer:** Thank you. We followed your protocol.
